# Environmental factors and hygiene behaviors associated with facial cleanliness and trachoma in Kongwa, Tanzania

**DOI:** 10.1371/journal.pntd.0009902

**Published:** 2021-10-28

**Authors:** Xinyi Chen, Beatriz Munoz, Meraf A. Wolle, Geordie Woods, Michelle Odonkor, Fahd Naufal, Harran Mkocha, Sheila K. West

**Affiliations:** 1 Dana Center for Preventive Ophthalmology, Wilmer Eye Institute, Johns Hopkins University, Baltimore, Maryland, United States of America; 2 Sightsavers, New Orleans, Louisiana, United States of America; 3 Kongwa Trachoma Project, Kongwa, United Republic of Tanzania; Fundacao Oswaldo Cruz, BRAZIL

## Abstract

**Background:**

Having a clean face is protective against trachoma. In the past, long distances to water were associated with unclean faces and increased trachoma. Other environmental factors have not been extensively explored. We need improved clarity on the environmental factors associated with facial cleanliness and trachoma prevalence, especially when the disease burden is low.

**Methodology/Principle findings:**

A cross-sectional survey focusing on household environments was conducted in all 92 villages in Kongwa, Tanzania, in a random selection of 1798 households. Children aged 0–5 years in these households were examined for facial cleanliness. In each of the 50 randomly-selected villages, 50 children aged 1–9 years were randomly selected and examined for trachoma. In a multivariate model adjusting for child age, we found that children were more likely to have clean faces if the house had a clean yard (OR 1.62, 95% CI 1.37–1.91), an improved latrine (OR 1.11, 95% CI 1.01–1.22), and greater water storage capacity (OR 1.02, 95% CI 1.00–1.04), and if there were clothes washed and drying around the house (OR 1.30, 95% CI 1.09–1.54). However, measures of crowding, wealth, time spent on obtaining water, or the availability of piped water was not associated with clean faces. Using a cleanliness index (clean yard, improved latrine, washing clothes, ≥1 child in the household having a clean face), the community prevalence of trachoma decreased with an increase in the average value of the index (OR 2.28, 95% CI 1.17–4.80).

**Conclusions/Significance:**

Access to water is no longer a significant limiting factor in children’s facial cleanliness in Kongwa. Instead, water storage capacity and the way that water is utilized are more important in facial cleanliness. A household cleanliness index with a holistic measure of household environment is associated with reduced community prevalence of trachoma.

## Author summary

Trachoma, a leading cause of blindness, is caused by repeated infection that is spread from person to person via contaminated ocular and nasal discharge. Having a clean face is protective against trachoma. In the past, long distances to water were associated with unclean faces. Other environmental elements have not been extensively explored. We need improved clarity on the environmental factors affecting facial cleanliness and trachoma prevalence, especially when the disease burden is low but still above 5%. We conducted a household survey in every village in Kongwa district, Tanzania, and found that access to water has significantly improved and is no longer a limiting factor in children’s facial cleanliness. When households have more storage capacity for water used for washing, wash their clothes frequently and clean their yards, young children in the households are more likely to have clean faces. We then created a household cleanliness index that comprises a clean yard, washed clothes, an improved latrine and at least one child in the household having a clean face. We found that an increased average index score in the community is associated with a decreased prevalence of trachoma, suggesting that a multi-pronged approach to improve household cleanliness is valuable.

## Introduction

Trachoma is the leading infectious cause of blindness worldwide, particularly affecting those living in low-resource areas [1]. Trachoma is the result of repeated infection with *Chlamydia trachomatis* (*C*. *trachomatis*), which causes conjunctivitis in young children [2], who are the main reservoirs of infection. *C*. *trachomatis* is transmitted in infected ocular and nasal discharge via direct contact, fomites, or eye-seeking flies [3–5].

Facial cleanliness is part of the multi-pronged approach known as the SAFE strategy to eliminate trachoma [1]. Studies have demonstrated the trachoma-protective effect of keeping children’s faces free of ocular and nasal secretions [6–14]. Washing faces as often as needed is apparently a crucial way to keep faces clean; current educational programs focus on the importance of face-washing [15–21]. However, further work to evaluate additional factors that influence facial cleanliness is needed. Infrastructure constraints such as distance to water may limit the ability to practice face-washing habits despite the knowledge to do so [22]. Family and societal value placed on water and hygiene may influence whether water is used for washing [23–25]. Additionally, there might be environmental elements that make children more prone to having unclean faces. In earlier studies in Tanzania, caregivers expressed frustration about the futility of washing children’s faces as they would soon become dirty again from interaction with a dirty environment [11,24]. A study found contamination of household environments including clothes, bedding, and furniture with *C*. *trachomatis* [5].

The purpose of the current study was to understand the effect of environmental factors and hygiene behaviors on children’s facial cleanliness in Kongwa district, Tanzania, where the overall trachoma prevalence was 7%. Once a district has reached low levels of trachoma, the factors affecting clean faces may change, and the relationship with trachoma may also change. We also sought to create a composite household cleanliness index and investigate its association with village-level trachoma prevalence.

## Methods

### Ethics statement

Approval for this study was granted from the Institutional Review Board of Johns Hopkins University School of Medicine and Tanzania National Institute for Medical Research. Formal verbal consent was obtained in local languages from all survey respondents and parents/guardians of the children. The study adhered to the tenants of the Declaration of Helsinki.

### Setting

The study was conducted in all 92 villages in Kongwa district in Tanzania. Research from 1986 showed that 80% of the households traveled over an hour one-way for water and the estimated prevalence of trachoma in pre-school children was 60% [23]. The district has since received multiple rounds of mass drug administration, most recently in 2016. The estimated prevalence of trachoma follicular (TF) in children aged 1–9 years in the district in 2017 was 7%.

### Household environmental survey

A standardized household environmental survey was conducted from May to September 2017. Eligible households had to have at least one child aged 0–5 years present. In each mtaa (geographic neighborhood) of a village in the district, 4 houses were randomly selected using a random-walk method. If the village had fewer than 5 mtaas, the number of houses per mtaa was increased so that at least 20 households per village were examined. Data were collected on reported water availability (water source, travel time to water source, wait time at the water source), observations of storage containers for water used for washing, observations on clothes washing, crowding (total number of people sleeping in the house at night, total number of children sleeping in the house at night, number of sleeping rooms), yard cleanliness (observed evidence of having been swept, presence of feces, garbage, and trash), observed latrine characteristics, and wealth parameters (ownership of lightbulb or radio). In the survey, garbage was defined as proteinaceous waste that could breed flies, and trash referred to paper or plastic litter.

### Facial cleanliness

All children aged 0–5 years in the sample households in the environmental survey were examined for facial cleanliness. The interviewers observed the presence of ocular and nasal discharge. Ocular discharge was defined as any fluid or dry matter on the lid margin or the lid (excluding tears). Nasal discharge was defined as wet or dry discharge visible outside the nostril when the child was examined in frontal view without being stared up the nostril. The face of a child was considered unclean if either ocular or nasal discharge was present [26]. The examiners also noted if the child’s face had just been washed by observing if there was water on the face and if the mother was currently washing the children’s faces.

### Trachoma prevalence

In October 2017, a separate trachoma survey was carried out in 50 randomly selected villages in Kongwa district. One grader examined 50 randomly selected children aged 1–9 years in each village. To examine the eyes, the grader everted both upper eyelids and scored for the presence of TF using the simplified WHO criteria [27]. TF was recorded as being present if it was seen in either eye.

### Data analyses

Questionnaire data were entered using a custom program in Access. Data were encrypted and sent to Johns Hopkins University for analyses. All data were analyzed using R version 4.0.0 [28].

The household water storage capacity was estimated by multiplying the number of containers to store water for washing by the size of the biggest container. The size of the biggest container was assigned “1” if it was the same size as a plastic bucket, “0.75” if smaller, or “1.25” if larger. The household water storage capacity is a composite variable with no units.

The one-way travel time for water source and the wait time for water at the source was assigned a categorical number: “1” if less than 30 minutes, “2” if 30 minutes to one hour, or “3” if more than one hour. We created a composite variable (no units): the total time spent on obtaining water. The total time spent on obtaining water = one-travel time x 2 + wait time.

A clean yard was defined as a binary variable (yes or no). Principal component analysis suggested that the first component consisted of having been swept, lack of garbage, and lack of trash; the variable “feces in the yard" was not included in the composite variable as it was only significant in the fifth component which explained 4.4% of the variance and it was rare among households. If a yard had been swept, lacked garbage, and lacked trash, then it was defined as a clean yard. If any of these criteria was not met, the yard was not considered a clean yard.

A numerical grade for latrine was assigned: 0 = no latrine, 1 = latrine with no improvements, 2 = latrine with a slab but no other improvements, 3 = improved latrine (latrine with slab and flush/pour flush or ventilated pit latrine). Household crowding was measured by the number of people or children sleeping in the house divided by the number of sleeping rooms.

The associations between continuous variables were evaluated using Spearman correlations. Comparisons between two groups of continuous variables were performed using Wilcoxon rank tests. A multivariate logistic regression analysis was used to model the probability of clean faces while adjusting for child age and household characteristics. A generalized estimating equation was used to account for clustering at the household level. The variables included in the model were either shown by previous studies or proposed in *a priori* hypotheses to be associated with clean faces.

Based on the *a priori* hypothesis that overall household attitude toward cleanliness is associated with trachoma, we created a household cleanliness index that was a summary score of the number of the following markers: a clean yard, clothes washed and drying around the house, an improved latrine, and at least one child in the household having a clean face. All four components in the summary score are binary (1 if yes, 0 if no). Thus, the cleanliness index has a score that ranges from 0 to 4. The association between the village-average household cleanliness score and TF prevalence (as categories of percentages) in the 50 villages was assessed using ordinal regression.

## Results

A total of 1798 households were surveyed, and 3084 children aged 0–5 years were examined for facial cleanliness. Overall, 1546 (50.1%) of the children had clean faces. Sixty-three percent of households had ≥1 child with a clean face (Table 1). Only 51 (1.7%) children had just washed their faces, so the observations on facial cleanliness were not skewed by recent face washing due to the presence of an interviewer. A slightly higher proportion of children under the age of 2 years old had clean faces than children over age 2 years (52.7% vs. 47.3%, *P* = 0.011). Over half of the households had a swept yard. A majority of households had an actively used latrine, although most of those were not improved.

**Table 1:** Characteristics of households (N = 1798) and children (N = 3084) in Kongwa survey in all 92 villages.

Characteristics	n (%)
*Water storage and retrieva*	
Average number of water storage containers/household	7.3 ± 4.1*
Households with the largest container of the following size Smaller than a plastic bucket Same as a plastic bucket Larger than a plastic bucket	23 (1.3) 869 (48.3) 898 (49.9)
Usual source of water (more than one is possible) Well or borehole Ground water or lake Piped water in village Buy from water sellers	88 (4.9) 428 (23.8) 1246 (69.3) 368 (20.5)
Households with a washstand at home	13 (0.7)
*Cleanliness of households*	
Households whose yard was swept	1150 (64.0)
Households whose front door area had visible feces	51 (2.8)
Households whose front door area had garbage	773 (43.0)
Households whose front door area had trash	859 (47.8)
Households with clothes washed and drying near the house	541 (30.1)
*Latrine*	
Households having an actively used latrine	1549 (86.2)
a pit latrine without a slab	1114 (62.0)
a pit latrine with a slab	98 (5.5)
an improved latrine (flush/ventilated with slab)	337 (18.7)
*Household crowding*	
Average number of people sleeping in each room	3.0 ± 0.9
Average number of children sleeping in each room	1.0 ± 0.6
Average number of sleeping rooms in a household	1.8 ± 0.8
*Wealth indicators*	
Households with a working radio at home	475 (26.4)
Households with a lightbulb in a socket at home	95 (5.3)
*Child characteristics*	
Average age of observed children (years)	2.4 ± 1.6
Female children	1575 (51.1)
Households with at least one child having clean faces	1135 (63.1)

* Mean ± standard deviation for continuous variables.

**Fig 1:**
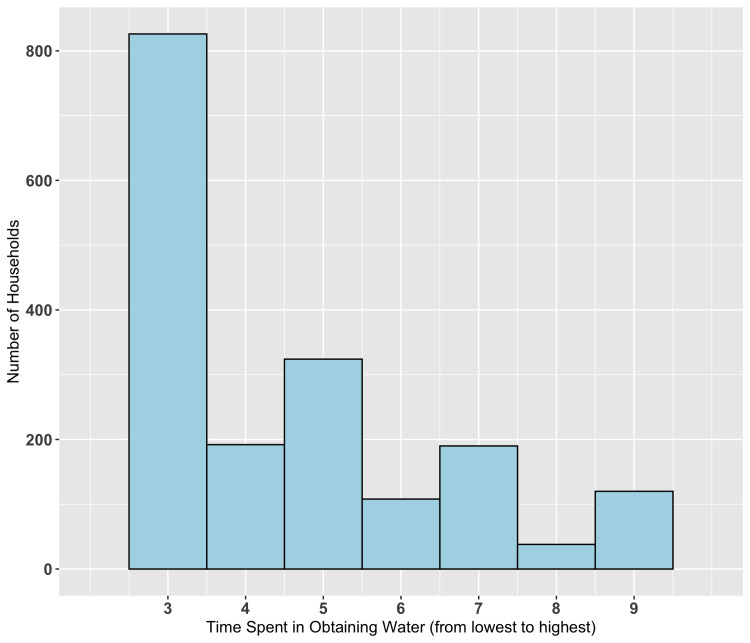
Distribution of total time spent obtaining water as a composite variable (no units). Total time spent obtaining water = one-way travel time x 2 + wait time, 1 for one-way travel time if < 30 minutes, 2 if 30 minutes– 1 hour, 3 if > 1 hour, with similar numerical assignments for wait time. For example, if time spent obtaining water = 9, the household was more than one hour’s walk from the water source and had to wait more than one hour for water once at the water source.

**Fig 2:**
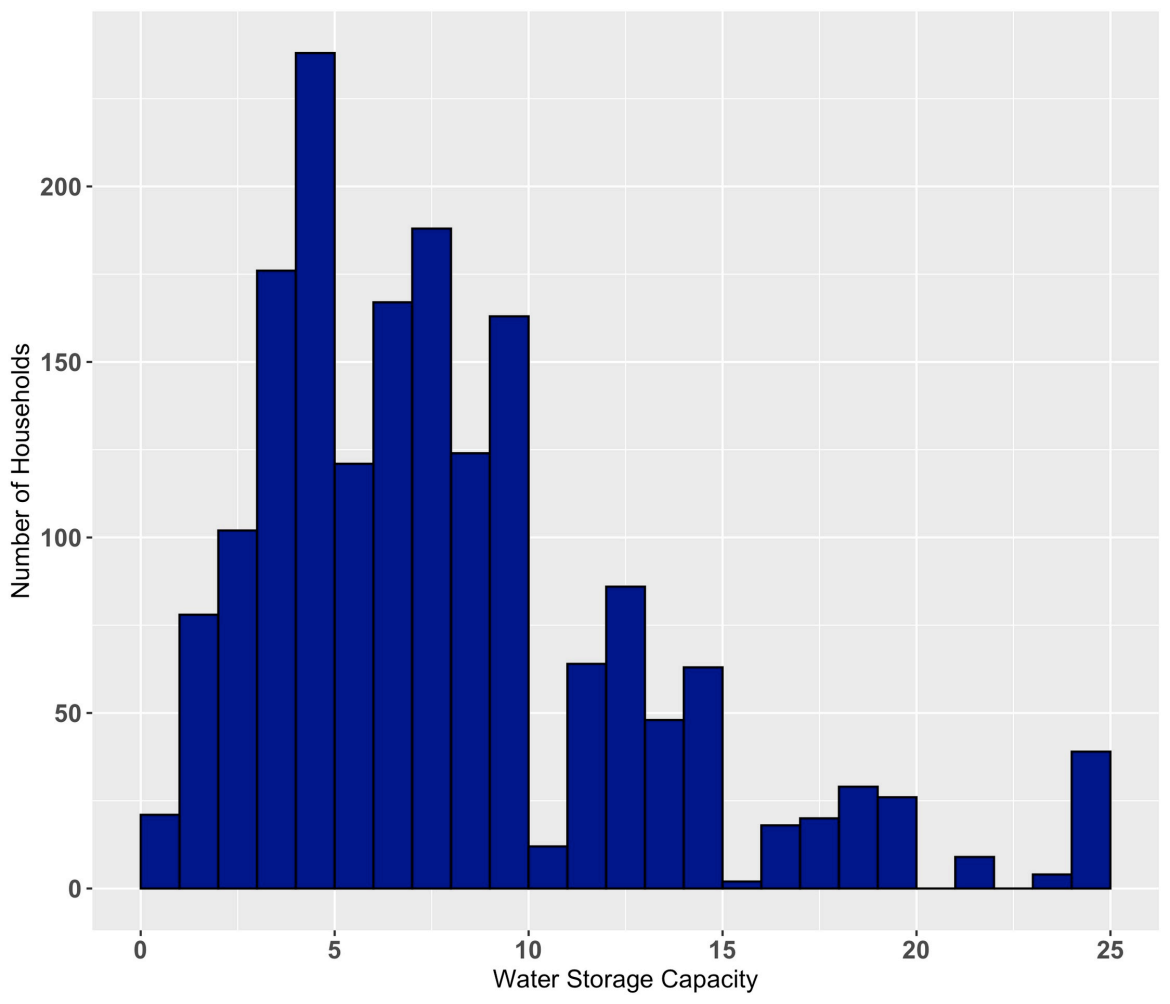
Distribution of household water storage capacity as a composite variable (no units). The bucket size was assigned 1 if the size of the largest of the containers was the same as a plastic bucket, 0.75 if smaller than a plastic bucket, or 1.25 if larger than a plastic bucket. Water storage capacity = the size of the largest bucket x number of water containers.

The most common water source was piped water. A total of 1239 households (68.9%) were less than 30 minutes’ walk from their usual water source, while 1008 households (56.1%) waited less than 30 minutes for water at the water source (Table 2). The distribution of total time spent on obtaining water is illustrated in Fig 1. The distribution of estimated household water storage capacity is shown in Fig 2.

**Table 2:** One-way travel time to water and wait time for water once at the water source.

	Wait time for water once at water source			*Total*
One-way travel time to water source	< 30 min	30 min– 1 hour	> 1 hour	
< 30 min	826 (45.9^a^)	192 (10.7)	221 (12.3)	1239 (68.9)
30 minutes– 1 hour	103 (5.7)	108 (6.0)	111 (6.2)	322 (17.9)
> 1 hour	79 (4.4)	38 (2.1)	120 (6.7)	237 (13.2)
*Total*	1008 (56.1)	338 (18.8)	452 (25.1)	1798 (100.0)

^a^ Numbers in parentheses indicate the percentage of the 1798 households.

There was no significant association between the total time spent obtaining water and household water storage capacity (correlation coefficient = -0.04, *P* = 0.119). There was also no correlation between the total time spent obtaining water and whether there were clothes washed and drying around the house (4.5 ± 1.8 for households with washed clothes, 4.6 ± 1.9 for households without washed clothes, *P* = 0.121). However, if a household had greater water storage capacity, they were more likely to have washed and drying clothes (8.9 ± 5.4 for households with washed clothes, 8.0 ± 4.8 for households without washed clothes, *P* = 0.002).

**Fig 3:**
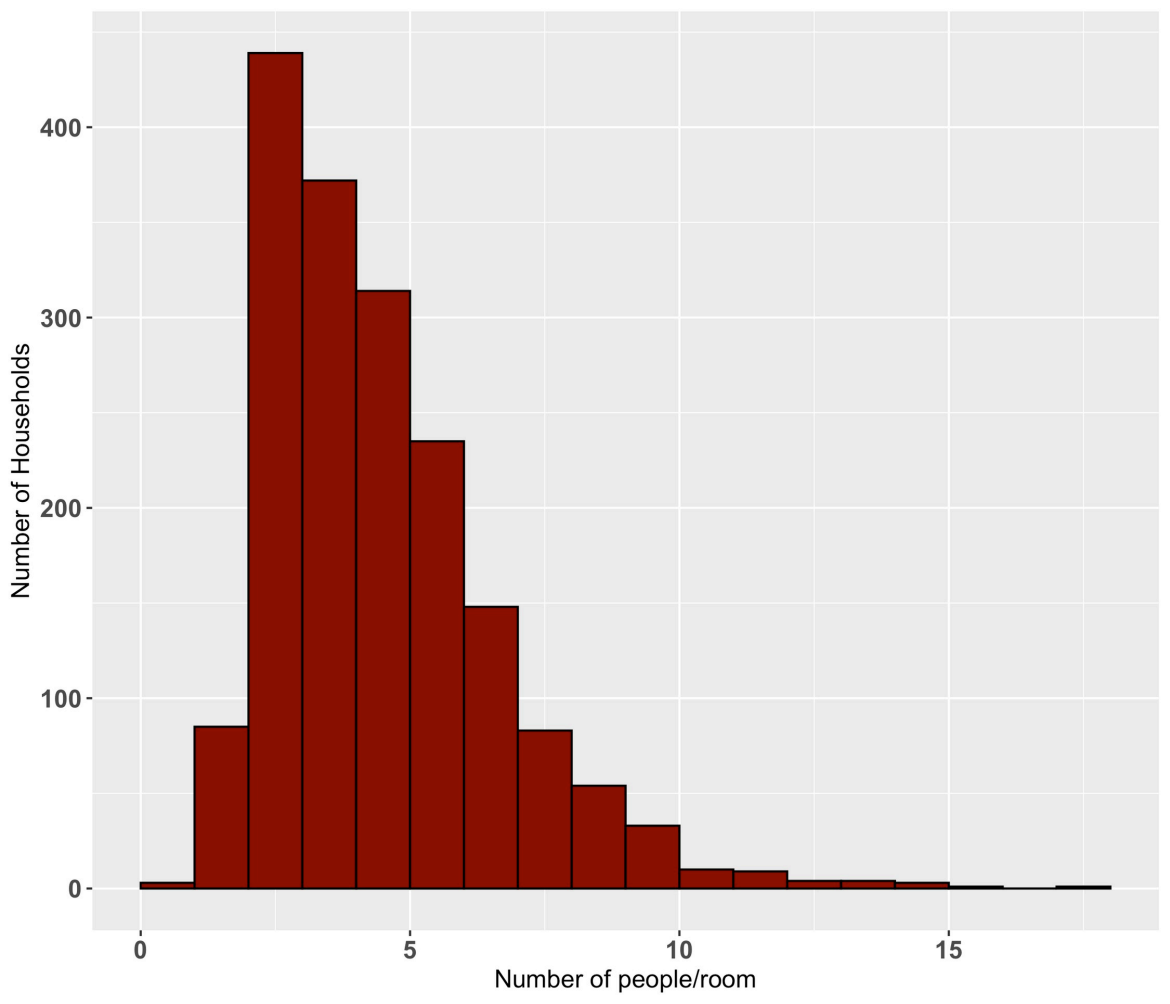
Distribution of the average number of people sleeping in each room at night (including both children and adults, calculated by the number of people sleeping in the house at night divided by the number of sleeping rooms in the house).

Household crowding was measured by the number of people sleeping in the house divided by the number of sleeping rooms, the distribution of which is shown in Fig 3.

The total time needed to obtain water in the household was not associated with children’s facial cleanliness (correlation coefficient = -0.007, *P* = 0.750). Simply owning a latrine was not correlated with clean faces (odds ratio = 0.84, 95% CI = 0.67–1.05). In the multivariate model, facial cleanliness in children was associated with increased water storage capacity, presence of washed clothes, younger child age, a higher grade of latrine, and a clean yard (Table 3). Wealth parameters, piped water, or crowded sleeping rooms was not associated with clean faces. We also performed analyses with the average number of children sleeping in each room and found no significant association either.

**Table 3:** Multivariate logistic regression of children’s facial cleanliness with generalized estimating equation to account for clustering at the household level.

	Odds Ratio (95% CI)	*P* value
Child age	0.91 (0.87, 0.96)	< 0.001
Water storage capacity	1.02 (1.00, 1.04)	0.038
Piped water	1.09 (0.91, 1.30)	0.349
Clothes drying	1.30 (1.09, 1.54)	0.003
Clean yard	1.62 (1.37, 1.91)	< 0.001
Latrine grade	1.11 (1.01, 1.22)	0.024
Average # of people sleeping in each room	1.00 (0.91, 1.09)	0.920
Ownership of either lightbulb or radio	1.12 (0.94, 1.35)	0.213

The household cleanliness index score was associated with our measure of wealth, i.e., ownership of a radio and/or a lightbulb. A total of 63.7% of the households having a radio/lightbulb had a cleanliness score of ≥2, compared to 44.6% of those without a radio/lightbulb (*P* < 0.001).

**Fig 4:**
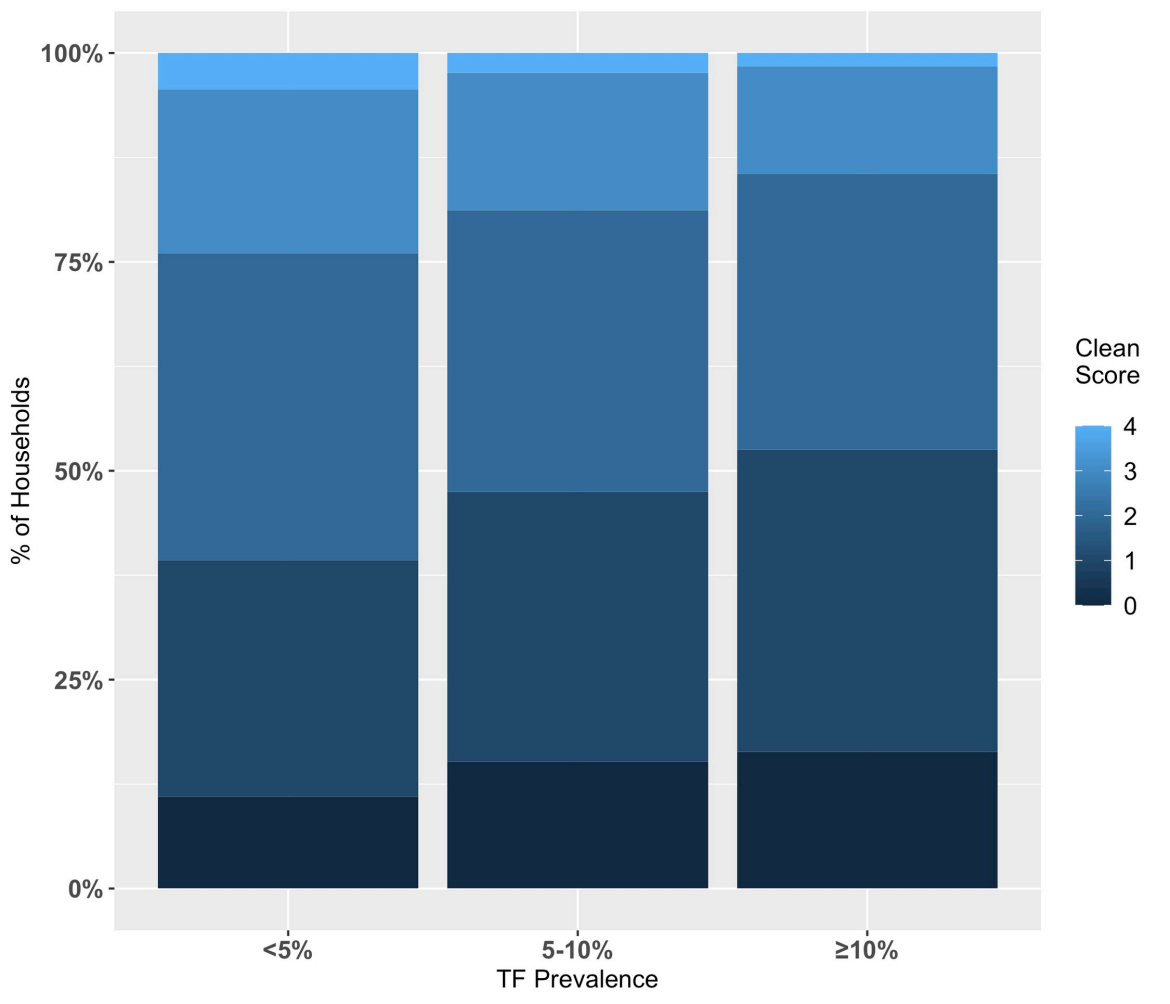
The percentage distribution of household cleanliness index scores according to community prevalence of trachoma. The household cleanliness index score is a summary score of the number of the following markers present and ranges from 0–4: a clean yard, clothes washed and drying around the house, an improved latrine, and at least one child in the household having a clean face. Each of the four components in the index is a binary variable (1 = yes, 0 = no).

The association of the household cleanliness index with the community trachoma prevalence was evaluated in the 50 communities that were part of the trachoma survey. When the community prevalence of trachoma was < 5%, over 50% of households had an index score of ≥ 2, whereas if the trachoma prevalence was ≥ 10%, less than 50% of households had a score of ≥ 2 (Fig 4). Using an ordinal regression model, we found that a 0.5-unit increase in the community average household cleanliness score was associated with a 2.28-fold increase in the odds of dropping the trachoma prevalence by one category (i.e., from ≥ 10% to 5–9.9%, or from 5–9.9% to < 5%; odds ratio = 2.28, 95% CI = 1.17–4.80). To ensure that it was not solely children’s clean faces that affected trachoma prevalence, we also did the above analyses with only the first three markers for cleanliness index and the relationships still held.

## Discussion

Our study identified several household environmental and hygiene factors associated with children’s facial cleanliness in Kongwa, Tanzania. We chose pre-school-age children rather than children aged 0–9 years so that we could observe children in their home environment rather than in school where a clean face is mandatory to be in classrooms.

Access to water is no longer a significant limiting factor in maintaining facial cleanliness in Kongwa, even in the dry season. This is in contrast to the 1986 survey, which found farther distance to water source to be a risk factor for unclean faces [23]. One possible reason is that most households now spend a short amount of time obtaining water, whereas in the 1986 survey, only 20.2% of the households travelled less than 30 minutes to reach their main water source. Moreover, 69.3% of the households in our study utilized piped water as their usual water source, which is an improved source [29]. Increased access to safely managed water is a prerequisite for any hygiene behavior intervention program. One of the limitations in previous trials of improving face-washing is the lack of water available for hygiene purposes [15,22].

Instead of access to water, household storage capacity for water that can be used for washing, which likely reflects the value placed on hygiene, was correlated with facial cleanliness. It appears that household and societal attitudes toward hygiene and clean faces are now important in decisions on water use [9,11,15,24,25].

We did not find a relationship between crowded sleeping conditions and unclean faces in children. Crowding has been linked to increased trachoma in Gambia, and an increased number of children in the household was associated with trachoma in Tanzania [30,31]. However, to our knowledge, the association between crowding and facial cleanliness in children has not been studied. It is possible that our measures of crowding were insensitive, as we only considered the number of people or children per room and did not ascertain how many beds there were in each room.

In our study, a higher grade of latrine was associated with clean faces. Simply owning a latrine was not significantly correlated with clean faces, which suggests that the quality of the latrine matters and may indicate a general household willingness to improve environmental conditions. Studies on the relationship between latrine type and children’s facial cleanliness are scarce, but many studies have found an association between latrines and lower risks of trachoma. In one study, a protective effect of toilets with flush against trachoma was found; interestingly, toilets without a flush were more strongly associated with increased trachoma than no toilet [32].

Washing clothes was associated with facial cleanliness. The association may be the result of simultaneous behaviors wherein the caretaker just takes the opportunity while washing clothes to wash the children as well. It may also reflect an attitude about overall cleanliness in the home environment that includes clean clothes as well as children with clean faces.

This idea that there are attributes of a clean environment that also contribute to cleaning children’s faces is further borne out by the finding that children who lived in a house with a clean yard were more likely to have clean faces. Since dirt on the face is not part of the criteria for a clean face, there is no obvious direct link between a household having an unclean yard and a child having an unclean face. Both might just be part of an overall approach to household cleanliness.

We constructed a measure of household cleanliness, an index that included a clean yard, at least 1 child with a clean face, clean clothes drying in the yard, and an improved latrine. This index was associated with our measure of wealth, ownership of a radio/lightbulb. The association might reflect available funds to improve the latrine. However, the association persisted even when latrine was removed from the index. We note that the rest of the parameters of the index, a clean yard, a clean face, and clean clothes, are achievable without wealth, suggesting that the wealth variable may be a proxy for another, unmeasured, variable.

We found that the average of household’s scores in a community was associated with the community prevalence of trachoma. The odds of a community having trachoma <5% or between 5–9.9% increased over two-fold if the average household score increased by 0.5. This finding suggests that a multi-pronged approach may be indicated to eliminate trachoma so that the focus is not solely on latrines or facial cleanliness but on the totality of a clean environment.

We acknowledge some limitations in our study. First, it was a cross-sectional study, so we cannot presume causality of any associations. A second limitation was the timing of the study, which was in the respiratory illness season, when children are more likely to have copious nasal discharge, and thus less likely to have a clean face at any one observation. Our study was based on a single observation of clean faces, and it is more likely that several observations spread over the day would better characterize the facial cleanliness status. While it is possible that the presence of researchers in the village may have generated momentary changes in household hygiene habits, we feel it is unlikely for several reasons. The random selection of households was made on the day of the survey, so no advance notice was provided. Particular households had no idea whether they would be included in the study beforehand, and with only four or so households per entire neighborhood, the likelihood of selection was low. We feel it is unlikely that there was rapid cleaning of the houses and children once the team arrived, as evidenced by the low number of children whose faces were being washed at the time of the survey. In addition, the households were only informed that observations would be made around the house, but they did not know which specific parameters were to be observed. Another limitation of the study was the use of the same examiner for facial cleanliness and household characteristics. However, the team was only informed of our general interest in household characteristics but not of any hypotheses linking facial cleanliness to household characteristics. There was no observer bias in the assessment of trachoma as the household survey and the trachoma survey were independent and conducted in different sets of households. That is also why we cannot link trachoma at the household level with the household characteristics observed; we only have the community-level prevalence.

This study has several strengths. First, all 92 villages in Kongwa were visited for the household environmental survey. This provides an invaluable snapshot of environmental conditions in Kongwa at a time when the rate of trachoma was low but not yet <5%. It strengthens the generalizability of our findings, especially at the district level. Second, many of the variables were based on actual observations instead of reports by households. For example, although more households reported having a latrine, we did not count it as an active latrine if the latrine did not have a path to it or clearly had not been used. The use of observational data removes household reporting bias in evaluating associations. Third, we are able to use prior data from Kongwa to note changes over time in variables such as access to water and the proportion of children with clean faces that may help guide future activities. Finally, by examining more holistically the characteristics of the household, we could construct a measure of cleanliness at the household level that has some validity towards reducing the risk of trachoma.

In summary, our study identified several environmental and hygiene factors associated with facial cleanliness in children. The associations suggest that facial cleanliness is part of a larger paradigm around household hygiene, and that these factors are important for decreasing the risk of trachoma, at least at the community level. The fact that households now have, in general, a large capacity to store water as well as improved access to water suggests that a clean environment is attainable. In fact, within the study area, significant numbers of households were able to achieve ≥2 of the factors in the cleanliness index, suggesting that these can be regarded as behavioral norms. While current efforts in SAFE promote facial cleanliness, and emphasize water and latrines for the environmental improvement part, a behavior change approach that considers a “clean environment” holistically may be beneficial.

## Supporting information

S1 Data Excel spreadsheet with raw data. (XLSX)
